# Evaluation of caffeic acid mucoadhesive tablets on minor recurrent aphthous stomatitis: a randomized, double-blind, placebo-controlled clinical trial

**DOI:** 10.1186/s12903-024-03936-0

**Published:** 2024-02-09

**Authors:** Maedeh Salehi, Majid Saeedi, Reza Negarandeh, Azin Savabi, Anahita Lotfizadeh, Abolfazl Hosseinnataj, Tahereh Molania

**Affiliations:** 1 Department of Oral Medicine, Dental Research Center, Mazandaran University of Medical Sciences, Sari, Iran; 2 Faculty of Dentistry, Mazandaran University of Medical Sciences, Sari, Iran; 3 Department of Pharmaceutics, Faculty of Pharmacy, Mazandaran University of Medical Sciences, Sari, Iran; 4 Student Research Committee, Department of Pharmaceutics, Faculty of Pharmacy, Mazandaran University of Medical Sciences, Sari, Iran; 5 Dentist, Sari, Mazandaran, Iran; 6 Department of Biostatistics, Faculty of health, Mazandaran University of Medical Sciences, Sari, Iran

**Keywords:** Caffeic acid, Inflammation, Mucoadhesive Patches, Oral aphthous lesions, Pain

## Abstract

**Background:**

Oral aphthous stomatitis is a chronic inflammatory condition. Numerous medications have been investigated to treat the symptoms of the disease. However, these days patients prefer herbal medicines due to lower side effects. Considering the anti-inflammatory, analgesic, and anti-oxidant properties of Caffeic acid and its few side effects, the aim of this study was to assess the impact of Caffeic acid on recurrent aphthous stomatitis (RAS). investigating the effect of caffeic acid mucoadhesive tablets on the size and pain intensity of the aphthous lesions.

**Methods:**

in this double-blinded clinical trial study, 47 patients who met the inclusion criteria were selected by convenient sampling method. The patients were assigned to two groups randomly; the control group (placebo recipients) and the intervention group (Caffeic acid recipients). Patients were followed up for 7 days following the intervention. The diameter of the inflammatory lesion was measured in millimeters, and the pain intensity was recorded based on the VAS scale (Visual Analogue Scale). This trial was approved by the medical ethics committee of Mazandaran University of Medical Sciences (Ethical code: IR.MAZUMS.REC.1401.261) and received IRCT code of IRCT20220815055700N1on 03/09/2022.

**Results:**

the diameter of the lesion in both groups decreased over time, and there was no significant difference between the intervention and control groups, except on the fifth day when the diameter of the lesion was significantly greater in the control group (*P* = 0.012). From the second day, the control group’s average pain intensity was significantly higher than the intervention group’s pain intensity (*P* < 0.05).

**Conclusions:**

when comparing mucoadhesive tablets containing Caffeic acid and placebo, the findings demonstrated that Caffeic acid has a significant efficacy in reducing aphthous lesions’ diameter and pain intensity of the patients and are suggested for palliative oral aphthous lesions treatment since they showed significant anti-inflammatory and analgesic effects on recurrent aphthous stomatitis.

## Background

The oral mucosa is affected by the chronic inflammatory condition known as recurrent aphthous stomatitis. It is characterized by excruciating oral lesions unrelated to a primary disease [1]. The incidence rate ranges from 5 to 60% in different communities; generally, it is more common among younger people, women, and people from higher socioeconomic levels [2]. Genetics, nutritional deficits, stress, and immunological dysfunction are typical etiological variables. Other factors include systemic disorders, hormonal imbalance, mechanical trauma, viral and bacterial infections, dietary allergies, vitamin and microelement shortages, hereditary predisposition, and stress [3, 4]. RAS (Recurrent Aphthous Stomatitis) lesions are recurrent; stress and anxiety play a crucial role in their development and recurrence [5, 6].

All of these factors have the potential to upset the body’s balance between oxidants and antioxidants, which would then lead to the production of free radicals. The oxidative stress brought on by increased free radical concentrations can impair immune system function, leading to cellular damage [6]. The most crucial free radicals in a biological system are those produced by oxygen [7]. Increased oxidative stress and an imbalance of oxidants and anti-oxidants are the primary causes of oral aphthous stomatitis. Oxidative stress develops when the intracellular levels of Reactive oxygen species (ROS) are higher than the normal levels produced intra or extra-cellular [8]. Both internal and external processes can produce free radicals. Endogenous generation of free radicals is caused by a variety of processes, including ischemia, infection, immune cell activation, inflammation, mental stress, cancer, excessive exercise, and aging. Exposure to environmental toxins, heavy metals (Pb, Cd, Fe, As, and Hg), specific medications (bleomycin, gentamycin, cyclosporine, and tacrolimus), cigarette, smoke, chemical solvents, cooking (smoked meat, fat, and used oil), radiations, and alcohol can result in the production of exogenous free radicals [9].

Numerous medications and techniques including laser therapy, a widely used treatment in craniofacial area, have been investigated to treat the symptoms of the disease because the cause is unknown. Multifocal treatment is used, and it varies depending on the risk factors. In all cases, treatment is symptomatic and aims to alleviate pain and aphthous inflammation [10–13].

Due to adverse side effects of pharmaceutical medicines, most patients choose herbal remedies [14]. Plants, food, and samples of propolis commonly include caffeic acid (3,4-dihydroxycinnamic acid), an essential phenolic compound, especially in the form of caffeic acid phenethyl ester (CAPE) [15]. This substance has neuroprotective, antibacterial, anti-inflammatory, cytotoxic, anti-chemotherapy, and anti-radiotherapy toxicity [16].

Some applications of this material in dentistry to date include the prevention of tooth decay, the reduction of chemotherapy-induced oral mucositis, the treatment of oral cancer, the management of gum and periodontal diseases, the inhibition of plaque, and anti-inflammatory properties [17]. Additionally, caffeic acid possesses biological effects that are anti-proliferative, anti-oxidant, anti-neoplastic, and antifungal [15, 18].

It has been demonstrated that Caffeic acid is a potent antioxidant that can neutralize ROS and protect cell membranes from lipid peroxidation. With a strong antioxidant impact and modest antibacterial activity, it is also a carcinogenic inhibitor. Along with many other advantages, it can also aid in preventing from atherosclerosis and cardiac problems [19]. According to research on the anti-inflammatory properties of this chemical, caffeic acid lowers the levels of interleukin-1beta, interleukin-6, and TNF-alpha [20].

Since caffeic acid contains anti-inflammatory and anti-oxidant characteristics and is a plant-based substance, it is safe, and no study has been done that has precisely examined the compound’s impact on recurrent aphthous stomatitis. This study aimed to assess the effects of caffeic acid on pain intensity, inflammatory lesion diameter, and the treatment duration of recurrent aphthous stomatitis lesions. By this study we are looking for introducing a novel treatment using a new medicine made of Caffeic acid, a natural substance whose anti-inflammatory and analgesic effects has been proven in the treatment of other medical and dental problems.

## Methods

This randomized double-blinded clinical trial is conducted based on CONSORT reporting guidelines [21] and was approved by the medical ethics committee of Mazandaran University of Medical Sciences (Ethical code: IR.MAZUMS.REC.1401.261). It also received IRCT code of IRCT20220815055700N1on 03/09/2022.

Before participating in the trial, all patients signed a consent form after receiving a complete description of the treatment process and any potential complications.

### Participants and eligibility criteria

According to the research done by Ghorbani et al. (2020), which the mean and standard deviation of the lesion diameter in the intervention group was 1.9 ± 1.3, and in the control group was 4.1 ± 2, with a confidence level of 95%, a test power of 90%, and for the two-way test, by using the formula for comparing the two averages in the G-power software; the sample size of the current study was calculated at 13 in each group, and taking into account a 50% decline, it was increased to 25 participants in each group (totally 50 participants) [22].

Initially, 50 participants were chosen using a convenient selection technique based on the inclusion criteria. To divide the samples into two groups, the blocking approach was applied. In each block, two patients were in the intervention group and two patients in the control group, making the blocks quadrable. Samples were allocated randomly using a blocking technique and random number generator software. The patients were chosen based on the inclusion criteria among those who were referred to the Mazandaran dental school with recurrent aphthous stomatitis in the age range of 20 to 40 and reported a history of mild aphthous lesions in parts like the lips and buccal mucosa. The patients were randomized into two groups at random: 25 patients in the intervention group and 25 patients in the control group. The chief nurse of the dental clinic registered the patients and administered the participants’ medication (either caffeic acid mucoadhesive tablets or a placebo), who was not an analyst or an evaluator. The intervention lasted seven days.

Patients with minor recurrent aphthous stomatitis, those with aphthous lesions in the lips or buccal mucosa (these areas are more accessible and less mobile), those not using dentures, not taking antibiotics, and overall good health are all required for inclusion in this study.

Patients who were pregnant, smoked, unable to use mucoadhesive tablets, had Behcet’s syndrome or other syndromes characterized by aphthous-like lesions, had myopathy or muscular disorders, had skin and mucosal auto-immune diseases, had liver failure, had urticaria or experienced skin and mucosal itching, or couldn’t participate in the study for personal or social reasons were all excluded from the study [23].

### Developing mucoadhesive tablets

Mucoadhesive tablets were produced in the laboratory of Mazandaran University of Medical Sciences (Faculty of Pharmacy). Mucoadhesive tablets were made by eccentric punching machine and by direct compression method. The mucoadhesive tablet formulation was prepared with a confirmed percentage of Caffeic acid as an active pharmaceutical ingredient (API), Carbopol as a bioadhesive and biocompatible polymer to adhere the tablets to the mucosa and to control the release of the active pharmaceutical ingredient, mannitol as a filler and sweetener, magnesium stearate was prepared as a lubricant and microcrystalline cellulose (Avicel®) was used as a diluent and anti-adhesion to improve the formulation.

### Study protocol

The evaluator and each patient in both groups were blinded in this investigation. The initial visit to the dental clinic was considered the baseline (day 0), and the patients were instructed to refer during the first 24 h following the appearance of aphthous lesions. Patients were first reassured of the project’s safety during the initial visit, and they were then requested to read, sign, and complete a questionnaire outlining their medical and dental histories. Notably, the examiner in this study was blinded and unaware of the medication provided to each patient. The first mucoadhesive tablet was applied by the examiner to the patient’s aphthous lesion during the same session. Three times a day, in the morning, noon, and evening, the patients were instructed to take the tablets. The mucoadhesive tablet consumption instruction was explained to them in detail, and they were urged to remove the tablet after 30 min and refrain from eating or drinking for 30 min. The same technique was carried out in the control group using a placebo, which comprised all the ingredients found in caffeic acid mucoadhesive tablets but not the caffeic acid that serves as their main component. Every patient was instructed to abstain from using further anti-inflammatory medications during the study.

The diameter of the lesions and the inflammatory halo around them were measured using a metal caliper at 0 (baseline), 3, 5, and 7 days later to assess the healing of the lesions.

Additionally, patients were asked to use the Visual Analogue Scale (VAS) to assess the severity of their discomfort. Zero on this scale indicates no pain, while ten indicates the most severe pain. The patients determined the point on this scale that best described their level of discomfort and estimated it using a numerical scale (for example, from 1 to 10). After every meal, patients completed the questionnaire three times a day, recording their VAS. Following the stimulation brought on by chewing, ingesting, and food particles, the pain increases. Patients were deemed to have improved when their pain score was 1 and the diameter of their lesions was smaller than 1 mm [23].

### Statistical analysis

The data were reported using descriptive indicators of frequency, mean, percentage, and standard deviation. Shapiro Wilk test was used to check the hypothesis of normal distribution of the data, and the results showed that the data did not follow the hypothesis of normal distribution. Chi-square (compare qualitative variables in two group), Mann-Whitney (compare non normal value in two group) and GEE tests (compare non normal value in different times) were used in the present study. All obtained data were entered into SPSS 24 software and analyzed. The significance level was considered less than 0.05.

## Results

**Fig. 1:**
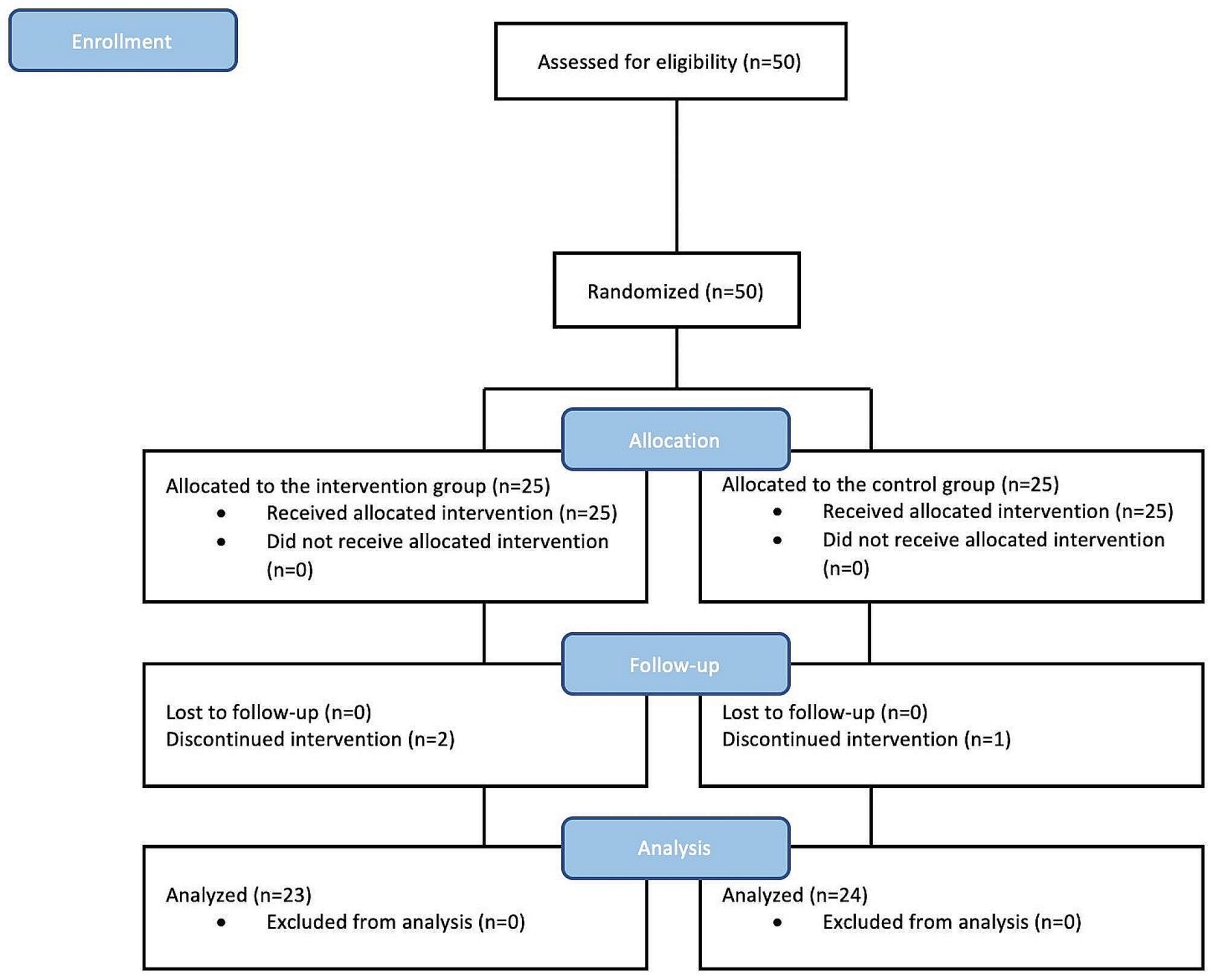
CONSORT flowchart

50 patients initially enrolled in the study; however, 47 patients continued to participate through the study’s conclusion and 3 participants withdrew in the middle of the study (Fig. 1). Participants didn’t claim any side effects related to the medicine.

The mean (standard deviation) age of the subjects was 29.70 ± 6.10; 6.4% had a history of systemic illnesses, and 3% of participants were female. Regarding age, gender, and history of systemic disorders, there was no discernible difference between the two groups (*P* > 0.05) (Table 1). As these variables did not have any statistically significant difference, we can perceive no confounding effect of these variables on the results of the present study (*P* > 0.05).

**Table 1:** Descriptive information of demographic variables in the intervention and control groups

Variable	Total	Intervention group	Control group	*p*-value
Age	29.70 ± 6.10	31.04 ± 6.75	28.42 ± 5.23	0.142^+^
Gender				> 0.999^++^
female	26 (55.3%)	13 (56.5%)	13 (54.2%)	
male	21 (44.7%)	10 (43.5%)	11 (45.8%)	
Systematic diseases				0.609^++^
No	44 (93.6%)	21 (91.3%)	23 (95.8%)	
Yes	3 (6.4%)	2 (8.7%)	1 (4.2%)	

+: Mann-Whitney test, ++: Cho-square test

Table 2 displays a statistically significant decreasing trend of lesion diameter in both groups when comparing intra-grouply from days 0 to 7 (*P* < 0.001). Compared to the control group, this trend was more pronounced in the intervention group. When comparing the groups, there was no noticeable difference in the lesion diameter on day 0. Greater lesion diameter decrease was seen in the intervention groups on days three and seven of the trial, though this difference was not statistically significant. On the 5th day of the trial, the mean lesion diameter of the treated group was significantly lower than the control group (*P* = 0.012).

**Table 2:** The inter-group and intra-group comparison of the average diameter of the lesions according to the days of study in the intervention and control groups

Variable	Day	Intervention	Placebo	*p*-value^+^	*p*-value^++^
lesion diameter	0	3.25 ± 1.98	2.57 ± 1.04	0.145	< 0.001
	3	2.11 ± 1.46	2.29 ± 1.40	0.663	
	5	0.79 ± 0.93	1.75 ± 1.50	0.012	
	7	0.22 ± 0.85	0.65 ± 0.90	0.094	
	*p*-value^++^	< 0.001	< 0.001		

+: Mann-Withney test ++:GEE (Generalized Estimated Equation)

**Fig. 2:**
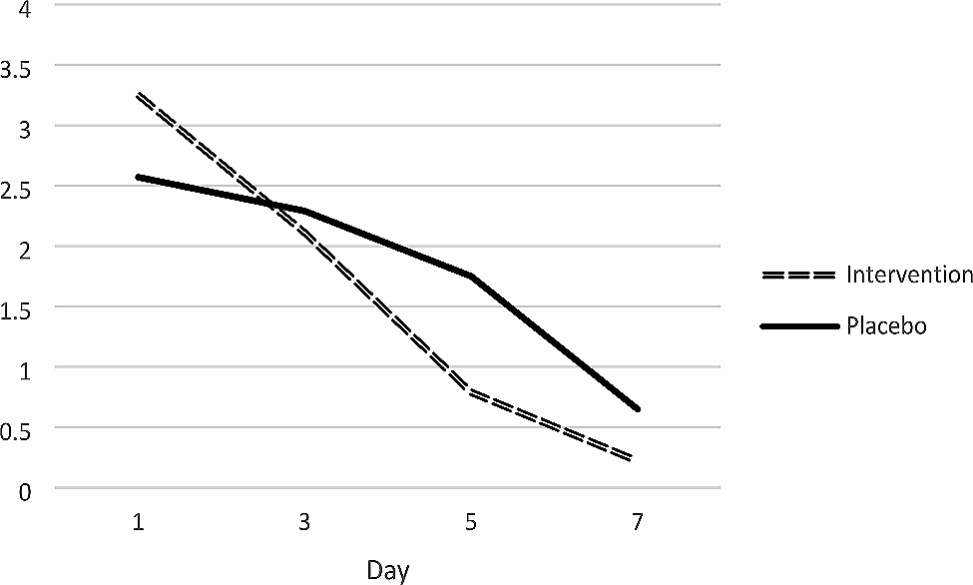
The intra-group and inter-group comparison of the lesions’ mean diameter showing a decreasing trend, which is more significant in the intervention group

Figure 2 demonstrates that the intervention group’s mean lesion diameter was greater than it was in the control group on day 0; However, this difference was not statistically significant. The lesion diameter decreased more noticeably in the intervention group compared to the control group.

**Fig. 3:**
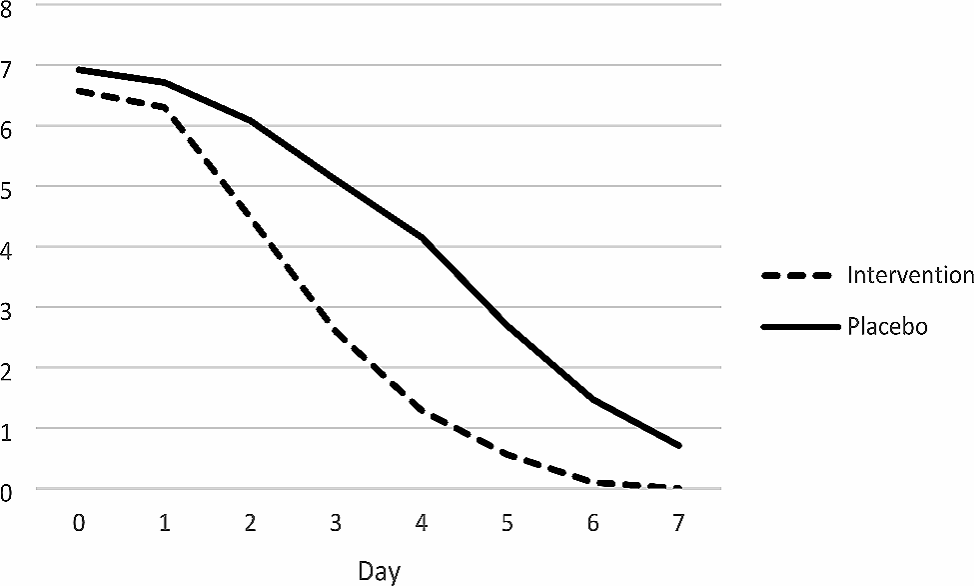
The average pain intensity of the patients showed a significant decreasing trend in both groups from day 2 to day 7, and was more significant in the intervention group

Table 3; Fig. 3 show that both groups’ mean pain intensity showed a significant decreasing trend (*P* < 0.001) in the intra-group comparison.

Pain intensity showed a significant decreasing trend in both groups from day 2 to day 7, with the intervention group showing a more significant decrease than the control group so that patients in the intervention group were deemed entirely improved by day 5.

**Table 3:** The inter-group and intra-group comparison of the pain intensity of the lesions according to the days of study in the intervention and control groups

Variable	Day	Intervention	Placebo	*p*-value^+^	*p*-value^++^
Pain intensity	0	6.57 ± 1.97	6.92 ± 2.32	0.579	< 0.001
	1	6.30 ± 1.94	6.71 ± 2.16	0.504	
	2	4.48 ± 2.13	6.08 ± 1.77	0.007	
	3	2.59 ± 2.04	5.10 ± 1.86	< 0.001	
	4	1.29 ± 1.78	4.15 ± 2.40	< 0.001	
	5	0.56 ± 0.95	2.69 ± 2.28	< 0.001	
	6	0.10 ± 0.29	1.47 ± 1.96	0.002	
	7	0.0 ± 0.0	0.71 ± 1.03	0.002	
	*p*-value^++^	< 0.001	< 0.001		

+: Mann-Withney test ++:GEE

## Discussion

This study aimed to determine the effect of caffeic acid mucoadhesive tablets on the size of the inflammatory lesion and the pain intensity associated with aphthous lesions in participants. The average diameter of the lesion in both groups’ intra-group examinations indicated a declining trend and a significant difference, according to the study’s findings. Except on the fifth day, the average diameter in the intergroup comparison did not reveal a statistically considerable difference.

Studies have revealed that caffeic acid enhances wound healing. Through anti-inflammatory mechanisms, this compound regulates neutrophil infiltration [24, 25]. Typically found in plants, meals, and propolis samples, caffeic acid is a phenolic molecule that is most commonly found as caffeic acid phenethyl ester (CAPE) [15].

As a secondary metabolite, caffeic acid phenethyl ester has anti-inflammatory therapeutic effects in the flavonoid form. Flavonoids have both analgesic and anti-inflammatory effects. The inhibition of the enzymes involved in generating inflammatory chemical mediators is the mechanism through which flavonoids work. Through the NF-κB pathway, CAPE, a substance in flavonoids, can block the synthesis of inflammatory cytokines such as TGF- β12, TNF-α, IL-6, and IL-1 [26, 27]. Numerous studies have investigated the anti-inflammatory, anti-oxidant, and analgesic capacities of caffeic acid to date. Still, it is impractical to compare the findings precisely because there hasn’t been any research on the use of caffeic acid mucoadhesive tablets for recurrent aphthous stomatitis treatment. Thus, we will review other studies on the anti-inflammatory and tissue repair capabilities of caffeic acid.

Puspasari et al. investigated the impact of topical caffeic acid phenethyl ester treatment on fibroblast expression and fibroblast growth factor-2 in diabetic traumatic wounds in the lower lip of rats. On days 5 and 7, the intervention group received topical treatment of caffeic acid phenethyl ester, which boosted the expression of FGF-2 and fibroblasts. Additionally, there was an association between higher fibroblast numbers and FGF-2 expression. This study showed that the local administration of caffeic acid phenethyl ester enhances fibroblast and FGF-2 expression during the healing of traumatic lesions [28].

In the study of Diab et al., the impact of a substance containing caffeic acid phenethyl ester on the healing and repairing of buccal mucosal wounds in the mouths of male rats showed that a group of mice that were treated with caffeic acid phenethyl ester had more epithelialization and collagen fibers formation than the control group [29].

The lesion size was assessed on days 1, 3, 5, and 8 in a trial that examined the impact of Proaftol (Extract Propolis 25%) mucoadhesive tablets on recurrent aphthous stomatitis, and the findings revealed a substantial decreasing trend. By the eighth day, the average of 5.7 mm from the first day had decreased to zero, signifying complete recovery [30]. Similar results were found in the current investigation, and on the seventh day, the average is almost zero. Even though caffeic acid is present in both formulations of these two mucoadhesive tablets, there may be a slight discrepancy in the results.

Ozório et al., Parolia et al. and, Wickiewicz et al. employed caffeic acid phenethyl ester-containing materials in the oral cavity as an intracanal medication and pulp covering agent in root canal therapy, wound healing, bone regeneration, and the construction of dental bridges. Their findings on wound healing and regeneration concurred with those of the present study [31–33].

The outcomes of the patient’s pain intensity from the first to seventh study days were another outcome. According to the findings, the mean pain intensity within each group significantly decreased in both groups. In the intergroup comparison, there was a notable difference in the two groups’ mean pain intensity from the second to the seventh day, with the control group experiencing more pain than the experimental group. Caffeic acid phenethyl ester was utilized by Moon et al. to promote wound healing and lessen post-tonsillectomy pain. The VAS scale measured pain severity on days 0, 1, 2, 3, and 7 to 10 following surgery. The caffeic acid phenethyl ester group experienced much less pain than the control group following tonsillectomy on days 3 and 7 to 10 [34]. As a result, their outcomes were in line with those of the current study.

Furthermore, the investigation of Proaftol (Propolis Extract 25%) mucoadhesive tablets on recurrent aphthous stomatitis and the pain it caused using the VAS scale on days 1, 3, 5, and 8 of the study shows a decreasing trend, so that on the first day, the average number was 3. On the eighth day, the number was zero, meaning the pain had utterly improved [30]. According to the VAS scale used in the current study, the intervention group’s pain level was zero on the seventh day.

Caffeic acid is also used for intrabuccal wound healing, epithelial repair following tooth extraction, and pain relief in the mouth. It has also effectively treated the painful dry socket problem following tooth extraction [35–38]. Anti-inflammatory, immune system balancing, and anti-oxidant characteristics are all possessed by caffeic acid. Additionally, this chemical lessens tissue damage brought on by oxidative stress and promotes epithelial regeneration during wound healing [39, 40]. The balance of oxidative and anti-oxidant components is generally necessary for the routine healing of wounds. Wound healing is hampered by higher levels of ROS and oxidative stress. By regulating this process, caffeic acid, as an anti-oxidant, enhances wound healing [41]. In addition, caffeic acid phenethyl ester has anti-bacterial effects and promotes wound healing and repairing by reducing the microorganisms’ growth [42]. As a result, it is anticipated that using caffeic acid mucoadhesive tablets will reduce the size of lesions, the intensity of pain, and discomfort in recurrent aphthous patients. This conclusion is based on the findings of the present study, the anti-inflammatory effect of caffeic acid, and the various properties of this substance that have been mentioned.

These days, treating aphthous lesions using mucoadhesive tablets impregnated with chemicals and herbal remedies is a widespread practice, and numerous studies have been done on this subject.

The use of mucoadhesive tablets made of medicinal components has been successful because they increase the medicine’s effectiveness and prolong its duration in the wound site [14, 23, 43]. The wound is protected by this treatment procedure, which lessens the lesion’s discomfort and suffering during the healing process. A secondary infection can be avoided, which lowers the requirement for antibiotics and antifungals [44]. It is hoped that mucoadhesive tablets containing caffeic acid will be used frequently as a treatment option in patients with recurrent aphthous stomatitis due to the positive results observed in their use. This is the first study examining the impact of caffeic acid mucoadhesive tablets on recurrent aphthous stomatitis. Thus, a thorough comparison of the findings was impossible. More examinations with greater sample sizes are needed. The lack of access to the patients during the study was another limitation.

## Conclusion

The findings of the present study demonstrated the noticeable effect of Caffeic acid on reducing the mean diameter of the aphthous lesions due to its wound healing property. Also, this substance reduced the pain intensity of the subjects. Thus, Caffeic acid mucoadhesive tablets are suggested for palliative treatment since they have anti-inflammatory and analgesic effects on recurrent aphthous stomatitis.as herbal-based medicines are mainly made of natural substances and have fewer side effects, patients’ tendency to these treatments is increasing and we believe that this medicine would also be welcomed. Furthermore, we suggest more studies on different form of this substance including its nanoform, applying them in the management of other oral diseases, and comparing them with the base form of the substance.

## Data Availability

All data generated or analyzed during this study are included in this published article.

## Abbreviations

VAS: Visual analogue scale
RAS: Recurrent aphthous stomatitis
ROS: Reactive oxygen species
CAPE: Caffeic acid phenethyl ester
API: Active pharmaceutical ingredient
GEE: Generalized estimated equation
